# Structure–Activity Relationship of Oligomeric Flavan-3-ols: Importance of the Upper-Unit B-ring Hydroxyl Groups in the Dimeric Structure for Strong Activities

**DOI:** 10.3390/molecules201018870

**Published:** 2015-10-16

**Authors:** Yoshitomo Hamada, Syota Takano, Yoshihiro Ayano, Masahiro Tokunaga, Takahiro Koashi, Syuhei Okamoto, Syoma Doi, Masahiko Ishida, Takashi Kawasaki, Masahiro Hamada, Noriyuki Nakajima, Akiko Saito

**Affiliations:** 1 Graduate School of Engineering, Osaka Electro-Communication University (OECU), 18-8 Hatsu-cho, Neyagawa-shi, Osaka 572-8530, Japan; E-Mails: me14a007@oecu.jp (Y.H.); me14a001@oecu.jp (Y.A.); me14a005@oecu.jp (T.K.); me12a003@oecu.jp (S.O.); me12a008@oecu.jp (S.D.); 2 Faculty of Engineering, Osaka Electro-Communication University (OECU), 18-8 Hatsu-cho, Neyagawa-shi, Osaka 572-8530, Japan; E-Mails: eg10a032@osakac.info (S.T.); eu11a066@oecu.jp (M.T.); eg10a006@osakac.info (M.I.); 3 Department of Pharmaceutical Sciences, Ritsumeikan University, 1-1-1 Nojihigashi, Kusatsu, Shiga 525-8577, Japan; E-Mail: kawa0227@fc.ritsumei.ac.jp; 4 Biotechnology Research Center and Department of Biotechnology, Toyama Prefectural University, 5180, Kurokawa, Imizu, Toyama 939-0398, Japan; E-Mail: hamada@pu-toyama.ac.jp

**Keywords:** condensed tannins, oligomeric flavonoid, synthesis, DPPH radical scavenging activity, antimicrobial activity, cancer cell proliferation inhibitory activity

## Abstract

Proanthocyanidins, which are composed of oligomeric flavan-3-ol units, are contained in various foodstuffs (e.g., fruits, vegetables, and drinks) and are strongly biologically active compounds. We investigated which element of the proanthocyanidin structure is primarily responsible for this functionality. In this study, we elucidate the importance of the upper-unit of 4–8 condensed dimeric flavan-3-ols for antimicrobial activity against *Saccharomyces cerevisiae* (*S. cerevisiae*) and cervical epithelioid carcinoma cell line HeLa S3 proliferation inhibitory activity. To clarify the important constituent unit of proanthocyanidin, we synthesized four dimeric compounds, (−)-epigallocatechin-[4,8]-(+)-catechin, (−)-epigallocatechin-[4,8]-(−)-epigallocatechin, (−)-epigallocatechin-[4,8]-(−)-epigallocatechin-3-*O*-gallate, and (+)-catechin-[4,8]-(−)-epigallocatechin and performed structure–activity relationship (SAR) studies. In addition to antimicrobial activity against *S. cerevisiae* and proliferation inhibitory activity on HeLa S3 cells, the correlation of 2,2-diphenyl-l-picrylhydrazyl radical scavenging activity with the number of phenolic hydroxyl groups was low. On the basis of the results of our SAR studies, we concluded that B-ring hydroxyl groups of the upper-unit of the dimer are crucially important for strong and effective activity.

## 1. Introduction

Currently, there is great interest in the strong biological activity of compounds commonly found in some foods. Such compounds have been considered safe functional compounds. Polyphenols are found in various plants such as vegetables and fruits and are consumed regularly [1,2]. Among polyphenol compounds, proanthocyanidins such as oligomeric flavan-3-ols and condensed tannins are known to be strongly bioactive compounds. It is widely believed that proanthocyanidins have a beneficial impact on health; therefore, they are included in various health foods, and scientific investigations of proanthocyanidins are becoming increasingly important. However, the structure–activity relationship (SAR) of proanthocyanidins is not well understood because, in many cases, they are obtained as mixtures of various analogues. Thus, purification of each compound is difficult. Therefore, synthesis is considered the most suitable method for clarifying the SAR of proanthocyanidins. Following contribution by Kozukowski *et al.* [3,4,5], many studies on proanthocyanidin synthesis and their biological properties were demonstrated [6,7,8,9,10,11,12,13,14].

**Figure 1:**
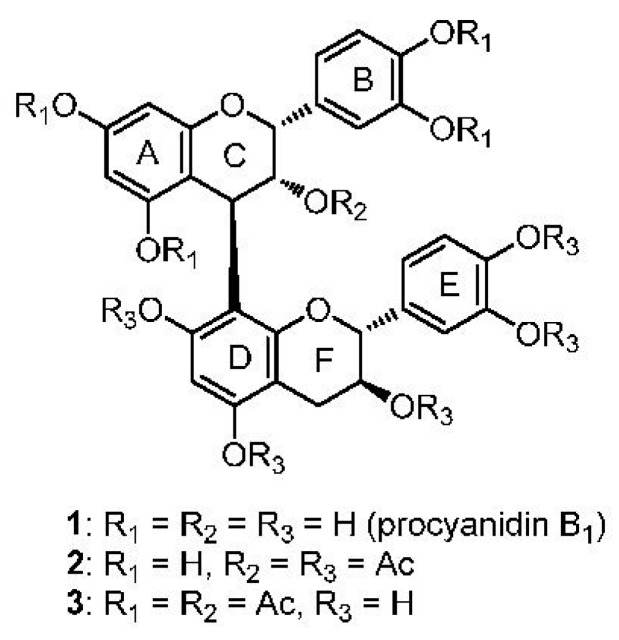
Structure of procyanidin B1 (**1**) and semi-acetylated analogues: lower-unit acetylated B1 (**2**) and upper-unit acetylated B1 (**3**).

We have also reported a simple, versatile, stereoselective and length controlled synthetic method for various procyanidins, a member of the proanthocyanidin class, which have two hydroxyl groups on the B-ring. We demonstrated that galloyl modification of the hydroxyl groups of flavan-3-ols enhanced their biological activities [15,16,17,18]. We further reported the synthesis of semi-acetylated analogues **1**–**3** of procyanidin B1, a dimeric flavan-3-ol, and discussed their inhibitory activities against HeLa S3 cell proliferation (Figure 1). The lower-unit acetylated procyanidin B1 (**2**) strongly inhibited proliferation of HeLa S3 cells, whereas procyanidin B1 (**1**) and the upper-unit acetylated analog **3** showed no inhibitory activity [19]. These results indicated that the upper-unit of dimeric flavan-3-ol is critical for biological activity. More recently, we elucidated that there is poor correlation between the inhibitory activity of HeLa S3 cell proliferation and 2,2-diphenyl-l-picrylhydrazyl (DPPH) radical scavenging activity [20]. In addition, we proved that the stereochemistry of the 3-hydroxyl group of flavan-3-ol-3,5-di-*O*-gallate is important for inhibitory activity against HeLa S3 cell proliferation [20].

**Figure 2:**
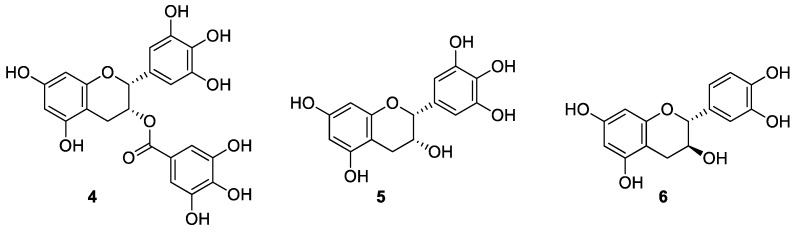
Structure of principal flavan-3-ols.

Here, we describe further investigations. Synthetic studies of dimeric flavan-3-ols, (−)-epigallocatechin-3-*O*-gallate (**4**), (−)-epigallocatechin (**5**), and (+)-catechin (**6**) as well as SAR studies were performed (Figure 2). In addition to the inhibitory activity assay against HeLa S3 cell, antimicrobial activity against *Saccharomyces cerevisiae* is also demonstrated. Oligomeric flavan-3-ols consisting of **4**, **5** and **6** are isolated from fermented foods such as beer, wine, *etc.* Flavan-3-ols and proanthocyanidins are known as antimicrobial active agents against various microorganisms, including yeast [21]. Although numerous studies about the biologically effect on yeast of plant extracts which are mixtures of various polyphenol compounds have been reported [22,23], there is little information allowing a detailed SAR study. Our results suggested that increases in the number of phenolic hydroxyl groups in the entire molecule correlate poorly with biological activities. In addition, we confirmed the importance of the upper-unit for the functionality of dimeric flavan-3-ols.

## 2. Results and Discussion

**Figure 3:**
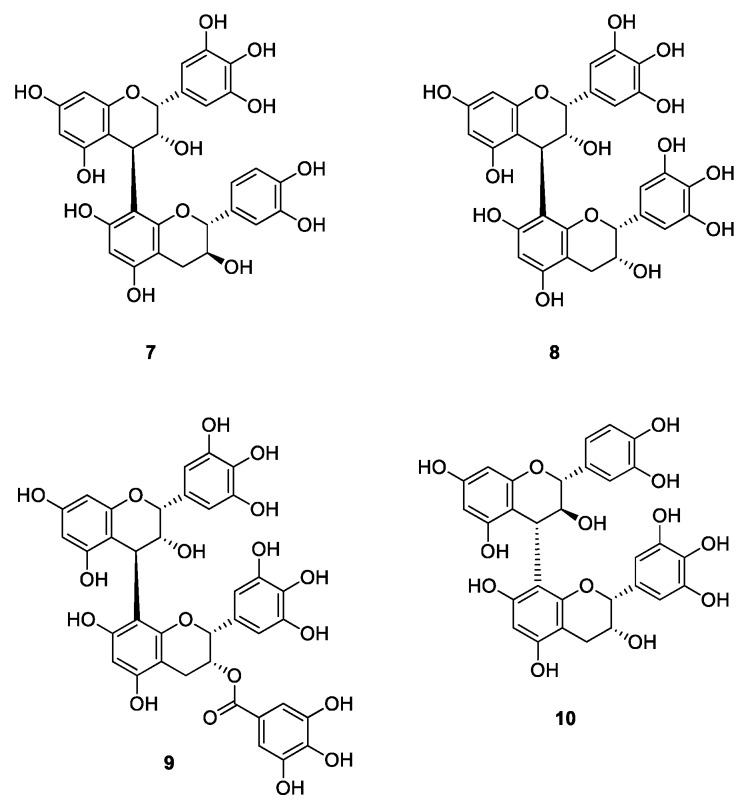
Synthesized dimeric flavan-3-ols.

The synthesis targets are shown in Figure 3. We synthesized four dimeric compounds: (−)-epigallocatechin-[4,8]-(+)-catechin (**7**), (−)-epigallocatechin-[4,8]-(−)-epigallocatechin (**8**), (−)-epigallocatechin-[4,8]-(−)-epigallocatechin-3-*O*-gallate (**9**), and (+)-catechin-[4,8]-(−)-epigallocatechin (**10**) to determine which part is the most important for biological activities and if the total number of phenolic hydroxyl groups in the entire molecule correlates with biological properties. The synthesized compounds **7**–**10** are natural products that can be isolated from beverages, e.g., green tea [24] and beer [25], and are known as strongly bioactive polyphenols.

**Scheme 1:**
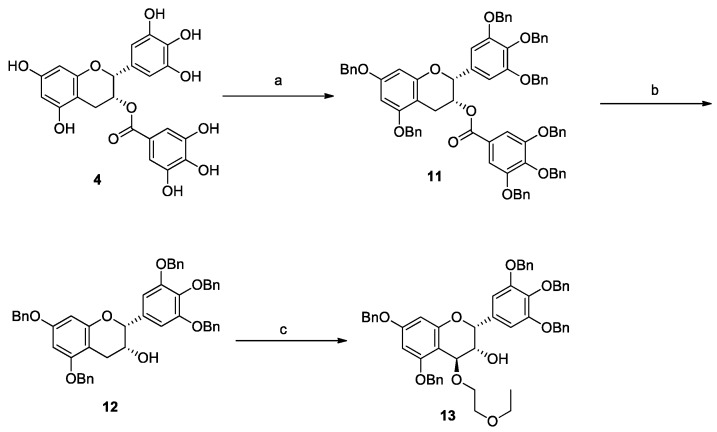
Synthesis of EGCG electrophile **13**. *Reagents and conditions*: (a) BnBr, K_2_CO_3_, DMF, 66%; (b) K_2_CO_3_, CH_2_Cl_2_–MeOH, quant.; (c) 2-ethoxyethanol, DDQ, CH_2_Cl_2_, 64%.

Compounds **7**–**9** were synthesized using an electrophile **13** derived from commercially available (−)-epigallocatechin-3-*O*-gallate (EGCG: **4**), as shown in Scheme 1. Eight phenolic hydroxyl groups of **4** were protected with benzyl groups. This reaction proceeded smoothly and afforded octa-*O*-benzylated EGCG (**11**) in 66% yield. The galloyl ester moiety of **11** was removed by alkaline hydrolysis to give **12** in quantitative yield. Compound **12** was converted to electrophile **13** by oxidative modification at the C4 position with DDQ and 2-ethoxyethanol [26].

**Scheme 2:**
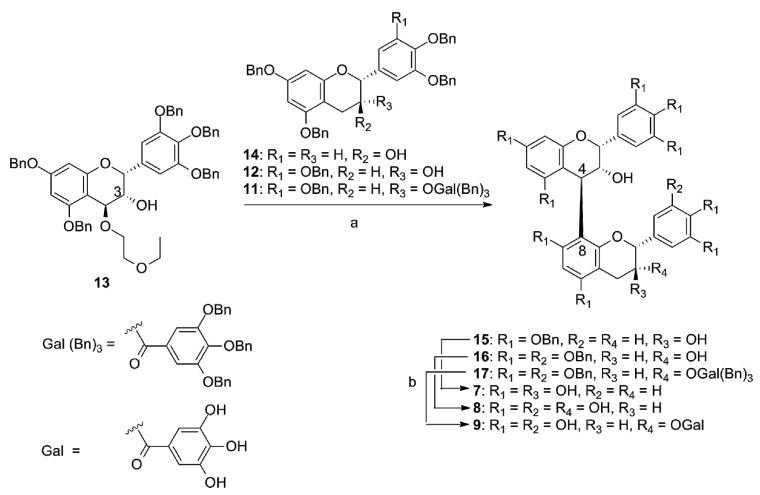
Synthesis of **7**–**9**. *Reagents and conditions*: (a) TMSOTf, −20 °C, CH_2_Cl_2_, **15**: 56%, **16**: 57%, **17**: 40%; (b) Pd(OH)_2_/C, H_2_, THF–MeOH–H_2_O then HPLC purification, **7**: 23%, **8**: 31%, **9**: 21%.

The electrophile **13** was condensed with **14**, **12**, and **11** and gave dimeric compounds **15**, **16**, and **17** in 56%, 57%, and 40% yields, respectively (Scheme 2). In those condensation reactions, only 3,4-*trans* products were obtained stereoselectively without 3-*O*-modification of electrophile **13**. Following HPLC purification, removal of benzyl groups by hydrogenation with Pd(OH)_2_/C under H_2_ atmosphere afforded **7**, **8**, and **9** as pure products in 23%, 31%, and 21% yields, respectively. We expected that comparison between **7** and **8** would show the effect on biological activities induced by the difference in the number of hydroxyl groups on the lower-unit B-ring and that the galloyl modified **9** could demonstrate whether the galloyl moiety of the lower-unit is effective to improve activity.

**Scheme 3:**
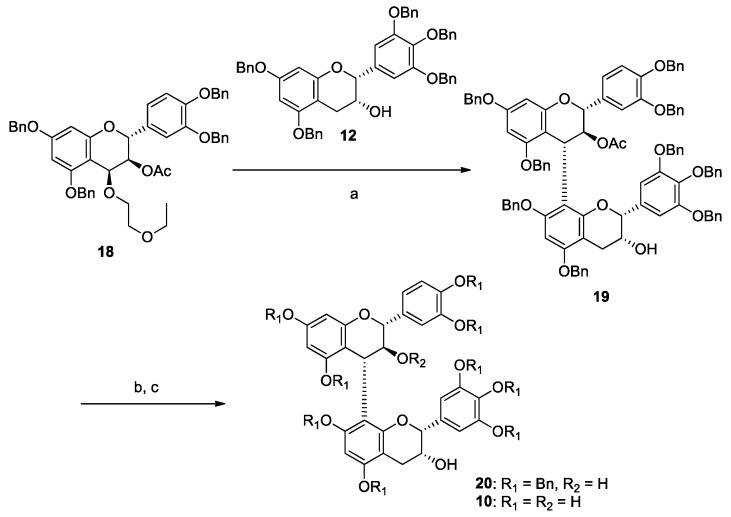
Synthesis of **10**. *Reagents and conditions*: (a) **12**, TMSOTf, CH_2_Cl_2_, −78 °C, 84%; (b) DIBAL-H, CH_2_Cl_2_, −78 °C, quant.; (c) Pd(OH)_2_/C, H_2_, THF–MeOH–H_2_O (20:1:1) then HPLC purification, 21%.

Synthesis of (+)-catechin-[4,8]-(−)-epigallocatechin (**10**), a reference compound of **7**, is shown in Scheme 3. The electrophile **18** [27] was condensed with a nucleophile **12** in the presence of TMSOTf as the Lewis acid at −78 °C in CH_2_Cl_2_ and gave dimeric compound **19** in 84% yield. The acetyl group of **19** was removed by DIBAL-H, and deprotection of the benzyl groups by hydrogenation conditions and HPLC purification afforded compound **10**, which had the same building unit as compound **7**.

### 2.1. DPPH Radical Scavenging Activity

**Figure 4:**
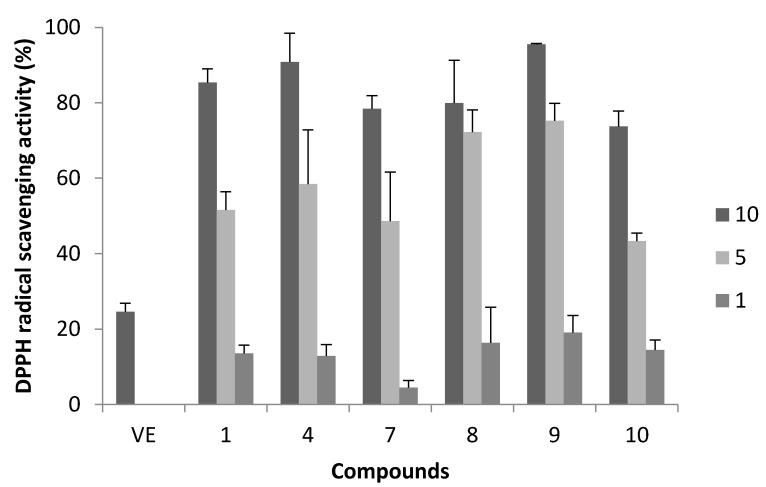
DPPH radical scavenging activity of synthesized compounds. 1 mL of 30 μM of DPPH solution in EtOH was added to 1 μL of sample solution in Dimethyl sulfoxide (DMSO) at final concentrations of 1, 5, and 10 μM (*n* = 6). Vitamin E was used as a control compound. Error bars represent standard deviation of the mean (*n* = 6).

Polyphenol compounds are known as extremely strong antioxidants and radical scavengers. In previous studies, we investigated the DPPH radical scavenging activity of synthesized oligomeric flavan-3-ols and derivatives [15,16,17,18,20]. In Figure 4, DPPH radical scavenging activities of **1**, **4**, **7**–**10** at a final concentration of 10, 5, and 1 μM are shown [28]. The SC_50_ values (the concentration of 50% scavenging activity) of these compounds were 6.0, 3.3, 4.7, 3.7, 2.9, and 5.9 μM, respectively. These SC_50_ values suggested that the number of phenolic hydroxyl groups was no correlated with DPPH radical scavenging activity. Surprisingly, EGCG (**4**), non-oligomeric compound, was a more effective radical scavenger than the oligomeric compounds.

### 2.2. Antimicrobial Activity against S. cerevisiae

**Figure 5:**
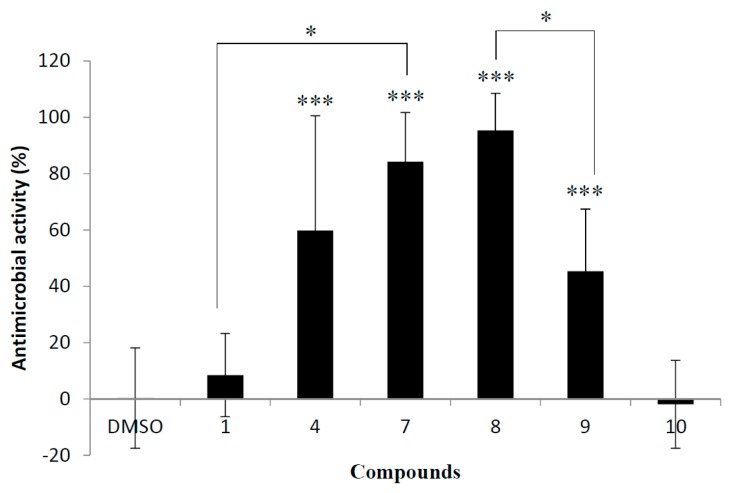
Antimicrobial activity of synthesized dimeric flavan-3-ols (**1**, **4**, and **7**–**10**) against *S. cerevisiae.* Comparison of antimicrobial activity (%) at final concentration of 50 μM after 20 h incubation. 200 μL of culture medium of *S. cerevisiae* at O.D._600nm_ 0.25 was treated with 1 μL of DMSO solution of **1**, **4**, and **7**–**10** at final concentrations of 50 μM. DMSO was used as a negative control. Error bars represent standard deviation of the mean (*n* = 6). *** *p* < 0.001 *vs.* DMSO-treated groups, asterisks indicate * *p* < 0.001 in Student’s *t* test.

Antimicrobial activity against *S. cerevisiae* is shown in Figure 5. Upper-unit epigallocatechin compounds **7** and **8** exhibited strong inhibitory activity against *S. cerevisiae* proliferation. It should be noted that activity comparison between compound **7** and reference procyanidin B1 (**1**) revealed that the three hydroxyl groups on the B-ring in the upper-unit are very important. Contrary to our expectations, it is clear from the activity between **8** and **9** that the galloyl moiety at the lower-unit resulted in decreased activity. Surprisingly, compound **10**, which had the same building unit (number of hydroxy groups) as compound **7**, showed no inhibitory activity of proliferation against *S. cerevisiae*. These data reveal that the existence of upper-unit hydroxyl groups in the dimeric structure is important for strong activities. Furthermore, microbial activity against *S. cerevisiae* showed no correlation to DPPH radical scavenger ability, which suggests that this antimicrobial activity is due to another mechanism.

### 2.3. Cervical Epithelioid Carcinoma Cell Line, HeLa S3 Proliferation Inhibitory Activity

**Figure 6:**
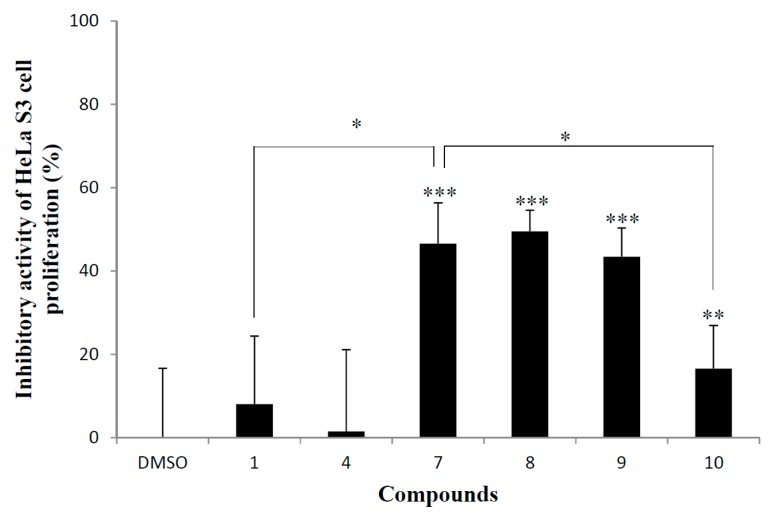
Inhibitory activity of synthetic dimeric flavan-3-ols against HeLa S3 cell proliferation. HeLa S3 was incubated with 1 μL of DMSO solution of each compound (final 100 μM) for 48 h. DMSO was used as a negative control. Error bars represent standard deviation of the mean (*n* = 14). *** *p* < 0.001, ** *p* < 0.005 *vs.* DMSO-treated groups, asterisks indicate * *p* < 0.001 in Student’s *t* test.

The inhibitory activities of the synthetic dimeric flavan-3-ols against HeLa S3 cell proliferation are shown in Figure 6. Similar to antimicrobial activity against *S. cerevisiae*, compound **10** showed low activity relative to other synthesized compounds **7**–**9**. Compared to compound **10**, the significance of the upper epigallocatechin unit of **7** is clear. Furthermore, the huge difference of activity between **1** and **7** indicates the importance of the third hydroxyl group on the B-ring. In addition, this activity was not enhanced by the introduction of a galloyl moiety to the lower-unit. Among the tested compounds, compound **7**, **8** and **9** showed comparable inhibitory activity, which indicates that the structure of the lower-unit is not significant.

## 3. Experimental Section

### 3.1. General Information

All commercially available chemicals for chemical synthesis were used without further purification. All reactions were performed under an argon atmosphere and monitored using thin-layer chromatography (TLC) with 0.25-mm pre-coated silica gel plates 60F254 (Art 5715, Merck KGaA, Darmstadt, Germany). An ATAGO (Minato, Japan) AP-300 spectrometer was used to measure optical rotation ^1^H- and ^13^C-NMR spectra (400/100 MHz) were recorded on a DD2 NMR Spectrometer (Agilent, Santa Clara, CA, USA). A microTOFfocus mass spectrometer (Bruker Daltonics, Billerica, MA, USA) was used to acquire electrospray ionization (ESI) mass spectra. The human cervical adenocarcinoma cell line, HeLa-S3, and the normal human fibroblast, WI-38 cell line, was provided by the RIKEN BRC through the National Bio-Resource Project of the MEXT (Tsukuba, Japan). Synthesized compounds were dissolved in dimethyl sulfoxide (DMSO) and stored at –25 °C. HPLC purification was carried out on an Ascentis^®^ column (SUPELCO^®^ analytical, Sigma Aldrich Co., St. Louis, MO, USA; 25 cm × 21.5 mm, 5 μm) using the solvents (A) 0.05% HCOOH in CH_3_CN and (B) 0.05% HCOOH and 10% CH_3_CN in H_2_O. Elution was done with a linear gradient 20%–100% B in 20 min (flow rate, 4.0 mL/min).

### 3.2. Synthesis

*(2R,3R)-Octa-O-benzyl-(*−*)-epigallocatechin 3-O-gallate* (**11**). To a solution of **4** (2.00 g, 4.36 mmol) in DMF (20.0 mL) was added K_2_CO_3_ (6.02 g, 43.6 mmol) and BnBr (6.22 mL, 52.4 mmol) slowly at 0 °C, and the mixture was stirred for 24 h. The reaction mixture was quenched with H_2_O and extracted with EtOAc and the organic phase was washed with brine and dried with MgSO_4_. Filtration, concentration, and silica gel column chromatography (*n*-hexane/EtOAc; 10:1*–*3:1, 40% CHCl_3_) afforded 3.39 g of **11** (2.88 mmol, 66%) as a colorless amorphous powder: ${\mathrm{[\alpha ]}}_{D}^{24}$ −76.8 (*c* 1.12, CHCl_3_); ^1^H*-*NMR (CDCl_3_) 7.43–7.10 (40H, m), 6.73 (4H, s), 6.40 (1H, s), 6.34 (1H, s), 5.66 (1H, br s), 5.05–4.59 (17H, m), 3.14–3.08 (2H, m); ^13^C-NMR (CDCl_3_) 164.8, 158.9, 158.0, 155.6, 152.9, 152.4, 142.7, 138.4, 137.8, 137.5, 136.9, 136.8, 136.4, 133.2, 128.6–127.2 (×19), 125.0, 109.2, 106.7, 101.0, 94.7, 94.0, 78.0, 75.1, 75.0, 71.2, 71.1, 70.2, 70.0, 68.3, 26.2; ESILRMS (*m*/*z*) 1205 (35), 1203 (82), 1202 ([M + Na]^+^, 100); ESIHRMS calcd. for C_78_H_66_O_11_Na, 1201.4503; found 1201.4514.

*(2R,3R)-Penta-O-benzyl-(*−*)-epigallocatechin* (**12**). To a solution of **11** (5.39 g, 4.57 mmol) in CH_2_Cl_2_/MeOH (1:1) was added K_2_CO_3_ (5.05 g, 36.5 mmol) at 0 °C, and the mixture was stirred for 30 min. The reaction mixture was quenched with saturated NH_4_Cl solution and extracted with EtOAc. Drying, filtration, concentration, and silica gel column chromatography (*n*-hexane/EtOAc; 10:1~2:1, 40% CHCl_3_) afforded 3.46 g of **12** (4.57 mmol, quant.) as a colorless amorphous powder: ${\mathrm{[\alpha ]}}_{D}^{24}$ −21.0 (*c* 1.38, CHCl_3_); ^1^H-NMR (CDCl_3_) 7.45–7.20 (25H, m), 6.82 (2H, s), 6.29 (2H, s), 5.14 (4H, s), 5.07 (2H, s), 5.02–4.90 (5H, m), 4.22 (1H, br s), 3.01 (1H, d, *J* = 13.6 Hz), 2.93 (1H, dd, *J* = 3.6, 13.6 Hz), 1.67 (1H, br); ^13^C-NMR (CDCl_3_) 158.3, 158.0, 155.1, 153.0, 138.4, 137.8, 137.0, 136.94, 136.91, 133.7, 128.60, 128.57, 128.53, 128.5 (C × 2), 128.2, 128.0, 127.91, 127.90, 127.8, 127.6 (C × 2), 127.5, 127.2, 106.2, 201.0, 94.7, 94.1, 78.6, 75.2, 71.3, 70.2, 70.0, 66.4, 28.1; ESILRMS (*m*/*z*) 781 (14), 780 (51), 779 ([M + Na]^+^, 100), 758 (2.1), 757 ([M + H]^+^, 3.7); ESIHRMS calcd. for C_50_H_44_O_7_Na, 779.2985; found 779.2975.

*(2R,3R,4S)-Penta-O-benzyl-4-(2″-ethoxyethoxy)-(*−*)-epigallocatechin* (**13**). To a solution of **12** (500 mg, 0.661 mmol) and ethoxyethanol (0.639 mL) in CH_2_Cl_2_ (35.0 mL) was added slowly DDQ (1.49 equiv., 223 mg, 0.982 mmol) at 0 °C. After stirring for 5 h at RT, excess of 4-(dimethylamino)pyridine was added to the solution at 0 °C and the mixture was stirred for 10 min. The resulting purple solid was removed by filtration and the filtrate was washed with water and brine, and dried (MgSO_4_). Filtration, concentration and short silica gel column chromatography (CHCl_3_) and silica gel column chromatography (*n*-hexane/EtOAc; 10:1–3:1, 20% CHCl_3_) afforded **13** (355 mg, 420 mmol, 64%) as a white foam: ${\mathrm{[\alpha ]}}_{D}^{28}$ −5.00 (*c* 0.40, CHCl_3_); ^1^H-NMR (CDCl_3_) 7.45–7.16 (25H, m), 6.85 (2H, s), 6.29 (1H, s), 6.28 (1H, s), 5.17 (1H, br s), 5.14 (4H, s), 5.06–5.02 (6H, m), 4.61 (1H, br s), 4.03–4.01 (1H, m), 3.85–3.75 (2H, m), 3.51 (2H, t, *J* = 5.2 Hz), 3.42 (2H, q, *J* = 6.8 Hz), 1.59−1.56 (1H, m), 1.17 (3H, t, *J* = 6.8 Hz); ^13^C-NMR (CDCl_3_) 160.6, 159.8, 156.1, 153.1 (×2), 138.4, 131.8, 136.9, 136.72, 136.70, 128.6 (×2), 128.5 (×2), 128.2, 128.0, 127.9, 127.8, 127.7, 127.6, 127.5, 106.4, 102.0, 94.4, 94.2, 75.2, 75.0, 71.4, 70.3, 70.1, 69.9, 68.9, 68.6, 66.5, 16.3; ESILRMS (*m*/*z*) 869 (19), 868 (62), 867 ([M + Na]^+^, 100), 846 (3.7), 845 ([M + H]^+^, 6.8); ESIHRMS calcd. for C_54_H_52_O_9_Na, 867.3509; found 867.3515.

*(2R,2″R,3R,3″S,4R)-[4,8]-2,3-cis-3,4-trans-Nona-O-benzyl-(*−*)-epigallocatechin-(+)-catechin* (**15**). To a solution of **13** (16.0 mg, 18.9 μmol) and **14** (55.0 mg, 84.5 μmol) in CH_2_Cl_2_ (5.0 mL) was added dropwise TMSOTf (50.0 μL, 25.0 μmol, 0.5 M solution in CH_2_Cl_2_) at −20 °C. After stirring for 5 min, the pale yellow reaction mixture was quenched with saturated NaHCO_3_. The mixture was extracted with CHCl_3_ and the organic phase was washed with water and brine, and dried (MgSO_4_). Filtration, concentration, and preparative silica gel TLC purification (hexane/EtOAc, 2.5:1) afforded 15.0 mg of **15** (10.5 μmol, 56%) as a colorless amorphous powder: ${\mathrm{[\alpha ]}}_{D}^{24}$ +30.0 (*c* 0.10, CHCl_3_); ^1^H-NMR (CDCl_3_, 0.77:0.23 mixture of rotational isomers) major isomer: 7.47–6.84 (34.65H, m), 6.80 (0.77H, t, *J* = 8.0 Hz), 6.73 (0.77H, d, *J* = 1.4 Hz), 6.50 (0.77H, dd, *J* = 1.4, 8.1 Hz), 6.37 (0.77H, s), 6.04 (0.77H, d, *J* = 2.2 Hz), 5.57 (0.77H, d, *J* = 2.2 Hz), 5.40 (0.77H, s), 5.18–4.91 (13.86H, m), 4.87 (0.77H, d, *J* = 5.2 Hz), 4.64 (0.77H, d, *J* = 11.2 Hz), 4.54 (0.77H, d, *J* = 11.3 Hz), 4.04 (0.77H, m), 3.76 (0.77H, ddd, *J* = 6.8, 9.2, 9.6 Hz), 3.65 (0.77H, d, *J* = 9.2 Hz), 3.26 (0.77H, dd, *J* = 6.6, 16.8 Hz), 2.60 (0.77H, dd, *J* = 9.6, 16.8 Hz), 1.76 (0.77H, br, s), 1.43 (0.77H, br s); minor isomer: 7.47–6.80 (11.27H, m), 6.24 (0.23H, d, *J* = 2.2 Hz), 6.21 (0.23H, d, *J* = 2.3 Hz), 6.10 (0.23H, d, *J* = 1.9 Hz), 5.31 (0.23H, s), 5.18–4.91 (4.83H, m), 4.42 (0.23H, d, *J* = 12.2 Hz), 3.61 (0.23H, m), 3.18 (0.23H, dd, *J* = 5.3, 16.2 Hz), 2.70 (0.23H, dd, *J* = 9.5, 16.3 Hz), 1.55 (0.46H, br s); ^13^C-NMR (CDCl_3_, 0.77:0.23 mixture of rotational isomers) major isomer: 158.2, 157.0, 156.0 (×2), 155.1, 154.6, 153.1 (×2), 149.3, 148.8, 138.3, 138.0, 137.4–137.0 (C × 8), 134.9, 130.3, 128.7–126.8 (C × 24), 120.6, 114.0, 112.1, 111.3, 106.3, 104.4, 104.2, 93.6, 93.1, 91.6, 81.7, 75.3, 72.4, 71.4 (×2), 71.3, 70.7, 70.3, 70.0, 69.6, 69.2, 68.7, 35.6, 29.1; minor isomer: 158.4, 157.8 (×2), 157.0, 156.0, 155.6, 152.8 (×2), 149.4, 149.0, 138.2, 138.0, 137.4–137.0 (C × 8), 134.8, 131.0, 128.7–126.8 (C × 24), 120.2, 115.0, 114.6, 113.0, 106.8, 102.8, 102.3, 94.5, 93.4, 92.8, 81.6, 75.6, 71.5 (×2), 71.3, 71.2 (×2), 71.0, 70.2, 68.4, 68.2, 35.7, 29.7; ESILRMS (*m*/*z*) 1431 (15), 1430 (43), 1429 (100), 1428 ([M + Na]^+^, 91), 1408 (11), 1407 (21), 1406 ([M + H]^+^, 22), 832 (9.4), 831 (15); ESIHRMS calcd. for C_93_H_80_O_13_Na, 1427.5497; found 1427.5455.

*(2R,2″R,3R,3″'R,4R)-[4,8]-2,3-cis-3,4-trans-Deca-O-benzyl-(*−*)-epigallocatechin-(*−*)-epigallocatechin* (**16**). To a solution of **13** (12.0 mg, 14.2 μmol) and **12** (42.1 mg, 55.7 μmol) in CH_2_Cl_2_ (5.0 mL) was added dropwise TMSOTf (40.0 μL, 20.0 μmol, 0.5 M solution in CH_2_Cl_2_) at −20 °C. After stirring for 5 min, the pale yellow reaction mixture was quenched with saturated NaHCO_3_. The mixture was extracted with CHCl_3_ and the organic phase was washed with water and brine, and dried (MgSO_4_). Filtration, concentration, and preparative silica gel TLC purification (hexane/EtOAc, 2:1) afforded 12.4 mg of **16** (8.09 μmol, 57%) as a colorless amorphous powder: ${\mathrm{[\alpha ]}}_{D}^{23}$ −30.0 (*c* 0.10, CHCl_3_) {lit. [26] ${\mathrm{[\alpha ]}}_{D}^{23}$ +16.6 (*c* 6.35, CHCl_3_)}; ^1^H-NMR (CDCl_3_, 0.75:0.25 mixture of rotational isomers) major isomer: 7.46–6.80 (37.5H, m), 6.89 (1.5H, s), 6.40 (1.5H, s), 6.03 (0.75H, d, *J* = 2.2 Hz), 5.71 (0.75H, d, *J* = 2.2 Hz), 5.56 (0.75H, s), 5.31–4.81 (15H, m), 4.68 (0.75H, d, *J* = 11.2 Hz), 4.52 (0.75H, d, *J* = 11.2 Hz), 4.08 (0.75H, br s), 3.98 (0.75H, s), 3.92–3.86 (0.75H, m), 3.01 (0.75H, d, *J* = 17.9 Hz), 2.90 (0.75H, dd, *J* = 4.8, 17.9 Hz), 1.77 (0.75H, d, *J* = 5.3 Hz), 1.51 (0.75H, d, *J* = 3.3 Hz); minor isomer: 7.46–6.80 (12.75H, m), 6.68 (0.25H, d, *J* = 1.9 Hz), 6.37 (0.5H, s), 6.26 (0.25H, d, *J* = 2.3 Hz), 6.21 (0.25H, s), 6.10 (0.25H, d, *J* = 2.3 Hz), 5.31–4.81 (5H, m), 4.63 (0.25H, d, *J* = 12.0 Hz), 4.39 (0.25H, d, *J* = 12.0 Hz), 4.09–3.85 (0.5H, m), 3.60–3.70 (0.25H, m), 3.12–2.98 (0.5H, m), 1.69 (0.25H, d, *J* = 6.0 Hz), 1.32 (0.25H, d, *J* = 7.5 Hz); ^13^C-NMR (CDCl_3_) 158.3, 158.0, 156.6, 156.0, 155.0, 154.4, 153.1, 152.6, 138.2, 138.0, 138.0, 137.6, 137.4, 137.3, 137.2, 137.17, 137.06, 137.02, 134.7, 133.5, 128.7, 128.6, 128.5, 128.42, 128.38, 128.32, 128.29, 128.14, 128.13, 128.02, 127.9, 127.8, 127.71, 127.70, 127.66, 127.63, 127.60, 127.4, 127.16, 127.14, 127.12, 126.8, 111.3, 106.1, 105.3, 104.3, 102.4, 93.7, 93.4, 91.6, 79.2, 75.8, 75.23, 75.22, 72.6, 71.5, 71.3, 71.1, 70.5, 70.4, 70.1, 70.0, 69.1, 66.4, 35.6, 28.5 (peaks of minor isomer were not identified); ESILRMS (*m*/*z*) 1537 (17), 1536 (52), 1535 ([M + Na]^+^, 100), 1534 (8), 1512 ([M + H]^+^, 0.8); ESIHRMS calcd. for C_100_H_86_O_14_Na, 1533.5915; found 1533.5861.

*(2R,2''R,3R,3''R,4R)-[4,8]-2,3-cis-3,4-trans-Trideca-O-benzyl-(*−*)-epigallocatechin-(*−*)-epigallo-catechin-3''-gallate* (**17**). To a solution of **13** (13.0 mg, 15.4 μmol) and **11** (81.3 mg, 69.0 μmol) in CH_2_Cl_2_ (5.0 mL) was added dropwise TMSOTf (40.0 μL, 20.0 μmol, 0.5 μM solution in CH_2_Cl_2_) at −20 °C. After stirring for 5 min, the pale yellow reaction mixture was quenched with saturated NaHCO_3_. The mixture was extracted with CHCl_3_ and the organic phase was washed with water and brine, and dried (MgSO_4_). Filtration, concentration, and preparative silica gel TLC purification (hexane/EtOAc, 2:1) afforded 12.0 mg of **17** (6.14 μmol, 40%) as a colorless amorphous powder: ${\mathrm{[\alpha ]}}_{D}^{25}$ −50.0 (*c* 0.10, CHCl_3_); ^1^H-NMR (CDCl_3_) 7.44–6.49 (66.5H, m), 6.94 (1H, s), 6.82 (1H, s), 6.79 (1H, s), 6.49 (1H, s), 6.44 (0.5H, s), 6.24 (0.5H, s), 6.13 (0.5H, br s), 6.12 (0.5H, br s), 6.06 (0.5H, br s), 5.82–5.78 (0.5H, m), 5.75 (0.5H, br s), 5.69 (0.5H, s), 5.43–5.38 (0.5H, m), 5.32–4.20 (25H, m), 4.26–4.22 (0.5H, m), 4.12–4.09 (0.5H, m), 3.36–3.30 (2H, m), 3.24 (0.5H, dd, *J* = 5.8, 18.3 Hz), 3.01 (0.5H, d, *J* = 18.3 Hz), 1.85 (0.5H, d, *J* = 6.4 Hz), 1.73 (0.5H, d, *J* = 4.5 Hz); ^13^C-NMR (CDCl_3_, 0.5:0.5 mixture of rotational isomers) 165.7, 165.4, 158.5, 158.3, 158.1, 157.2, 156.8, 156.5, 156.3, 156.2, 155.2, 155.0, 154.9, 155.6, 153.1, 153.0, 152.5, 152.4, 152.1, 143.0, 142.4, 138.4, 138.2, 138.04, 138.00, 137.86, 138.84, 137.7, 137.5, 137.4, 137.3, 137.2, 137.1, 137.0, 136.93, 136.91, 136.88, 136.82, 136.7 (×2), 136.6, 136.34, 136.28, 134.5, 134.4, 133.2, 132.7, 130.9, 128.73, 128.69, 128.60, 128.54, 128.47, 128.41, 128.39, 128.36, 128.34, 128.29, 128.28, 128.26, 128.25, 128.20, 128.18, 128.14, 128.10, 128.08, 128.07, 128.05, 128.03, 128.00, 127.96, 127.93, 127.88, 127.85, 127.78, 127.75, 127.72, 127.69, 127.65, 127.62, 127.59, 127.54, 127.46, 127.44, 127.42, 127.39, 127.32, 127.18, 127.17, 125.1, 124.7, 111.9, 111.3, 109.4, 109.0, 106.8, 106.3, 105.7, 104.7, 104.2, 103.1, 101.9, 94.8, 94.1, 93.7, 93.4, 92.4, 92.0, 78.6, 76.0, 75.6, 75.3, 75.2, 75.1, 75.0, 74.0, 72.7, 72.4, 71.5, 71.4, 71.33, 71.26, 71.1, 70.9, 70.73, 70.66, 70.1, 70.0, 69.9, 69.8, 69.3, 69.1, 68.2, 67.9, 37.1, 35.5, 29.7, 26.7 (22 peaks of benzyl groups were not observed); ESILRMS (*m*/*z*) 1958 (13), 1957 (18), 1956 ([M + Na]^+^, 13), 1351 (17), 1350 (25), 1235 (17), 1234 (24), 1187 (19), 1186 (32), 1177 (19), 1176 (26), 1143 (23), 1142 (40), 1099 (34), 1098 (58), 1011 (47), 1010 (100); ESIHRMS calcd. for C_128_H_108_O_18_Na, 1955.7433; found 1955.7427.

*(2R,2''R,3R,3''S,4R)-[4,8]-2,3-cis-3,4-trans-(*−*)-Epigallocatechin-(+)-catechin* (**7**). A solution of **15** (110 mg, 77.1 μmol) in THF/MeOH/H_2_O (20:1:1, 22 mL) was added to the mixture and hydrogenated over 20% Pd(OH)_2_/C (5 mg) under H_2_ atmosphere for 12 h at RT. Filtration and concentration afforded a pale brown solid, which was purified with HPLC purification to give 10.4 mg of pure **7** (17.5 μmol, 23%) as a pale brown powder: ${\mathrm{[\alpha ]}}_{D}^{24}$ +67.3 (*c* 0.033, MeOH) {lit. [26] ${\mathrm{[\alpha ]}}_{D}^{22}$ +24.0 (*c* 0.50, MeOH)}; ^1^H-NMR (CD_3_OD, −55 °C) 6.88 (1H, d, *J* = 8.6 Hz), 6.79 (1H, s), 6.67 (1H, d, *J* = 8.2 Hz), 6.35 (2H, s), 5.89 (1H, d, *J* = 2.2 Hz), 5.87 (1H, s), 5.80 (1H, s), 5.03 (1H, s), 4.99 (1H, d, *J* = 4.3 Hz), 4.64 (1H, s), 4.22–4.14 (1H, m), 3.93 (1H, s), 2.63–2.42 (2H, m); ^13^C-NMR (CD_3_OD, *−*55 °C) 158.5, 157.7, 156.4, 155.7, 153.6, 146.3, 146.0, 145.6, 132.9, 132.1, 131.8, 119.0, 115.8, 113.4, 107.6, 106.3, 106.1, 102.3, 99.6, 96.4, 95.4, 94.6, 81.2, 76.8, 72.8, 67.9, 36.5, 26.0; ESILRMS (*m*/*z*) 619 (13), 618 (48), 617 ([M + Na]^+^, 100), 597 (11), 596 (38), 595 ([M + H]^+^, 81); ESIHRMS calcd. for C_30_H_27_O_13_, 595.1452; found 595.1441.

*(2R,2''R,3R,3''R,4R)-[4,8]-2,3-cis-3,4-trans-(*−*)-Epigallocatechin-(*−*)-epigallocatechin* (**8**). A solution of **16** (95.0 mg, 61.9 μmol) in THF/MeOH/H_2_O (20:1:1, 22 mL) was added to the mixture and hydrogenated over 20% Pd(OH)_2_/C (5 mg) under H_2_ atmosphere for 12 h at RT. Filtration and concentration afforded a pale brown solid, which was purified with HPLC purification to give 11.7 mg of pure **8** (19.1 μmol, 31%) as a pale brown powder: ${\mathrm{[\alpha ]}}_{D}^{24}$ +10.3 (*c* 0.29, MeOH) {lit. [26] ${\mathrm{[\alpha ]}}_{D}^{22}$ +24.0 (*c* 0.50, MeOH)}; ^1^H-NMR (CD_3_OD, *−*55 °C) 6.58 (2H, s), 6.30 (2H, s), 5.85 (1H, s), 5.55–5.40 (2H, m), 4.96 (1H, s), 4.86 (1H, s), 4.59 (1H, s), 4.24 (1H, br s), 3.78 (1H, s), 2.92 (1H, d, *J* = 16.5 Hz), 2.79 (1H, d, *J* = 16.5 Hz); ^13^C-NMR (CD_3_OD, *−*55 °C) 157.8, 157.7, 157.5, 156.65, 156.56, 154.5, 146.5, 146.3, 133.04, 133.02, 131.5, 131.1, 107.0, 106.1, 105.9, 102.1, 99.4, 96.5, 95.5, 95.2, 79.2, 76.8, 73.5, 66.9, 36.5, 30.2; ESILRMS (*m*/*z*) 634 (18), 633 ([M + Na]^+^, 55), 613 (7.8), 612 (32), 611 ([M + H]^+^, 100); ESIHRMS calcd. for C_30_H_27_O_14_, 611.1401; found 611.1356.

*(2R,2''R,3R,3''R,4R)-[4,8]-2,3-cis-3,4-trans-(*−*)-Epigallocatechin-(*−*)-epigallocatechin 3''-O-gallate* (**9**). A solution of **17** (150 mg, 76.0 μmol) in THF/MeOH/H_2_O (20:1:1, 22 mL) was added to the mixture and hydrogenated over 20% Pd(OH)_2_/C (5 mg) under H_2_ atmosphere for 12 h at RT. Filtration and concentration afforded a pale brown solid, which was purified with HPLC purification to give 12.1 mg of pure **6** (15.9 μmol, 21%) as a pale brown powder: ${\mathrm{[\alpha ]}}_{D}^{24}$ −64.3 (*c* 0.14, MeOH); ^1^H-NMR (CD_3_OD, *−*55 °C) 7.00 (2H, s), 6.60 (2H, s), 6.41 (2H, s), 6.00 (1H, s), 5.96 (1H, s), 5.88 (1H, s), 5.59 (1H, br s), 5.12 (1H, br s), 5.07 (1H, br s), 4.79 (1H, br s), 3.91 (1H, br s), 3.04 (1H, d, *J* = 15.8 Hz), 2.89 (1H, d, *J* = 15.8 Hz); ^13^C-NMR (CD_3_OD) 167.6, 158.2, 157.8, 157.7, 156.5, 155.8, 154.4, 146.4 (×2), 146.0, 139.6, 133.3, 133.1, 131.7, 130.5, 121.0, 109.9, 107.9, 106.2, 106.1, 102.2, 99.0, 96.6, 95.6, 95.2, 78.1, 77.0, 73.6, 69.3, 36.5, 26.8; ESILRMS (*m*/*z*) 787 (7.5), 786 (24), 785 ([M + Na]^+^, 53), 765 (13), 764 (44), 763 ([M + H]^+^, 100); ESIHRMS calcd. for C_37_H_31_O_18_, 763.1510; found 763.1532.

*(2R,2''R,3S,3''R,4S)-[4,8]-2,3-trans-3,4-trans-3-Acetoxy-nona-O-benzyl-(+)-catechin-(*−*)-epi-gallocatechin* (**19**). To a solution of **18** (281 mg, 360 μmol) and 1**2** (959 mg, 1.44 mmol) in CH_2_Cl_2_ (30 mL) was added dropwise TMSOTf (432 μL, 432 μmol, 1.0 M solution in CH_2_Cl_2_) at −78 °C. After stirring for 5 min, the pale yellow reaction mixture was quenched with saturated NaHCO_3_. The mixture was extracted with CHCl_3_ and the organic phase was washed with water and brine, and dried (MgSO_4_). Filtration, concentration, and silica gel column chromatography (*n*-hexane/EtOAc; 8:1*–*2:1, 40% CHCl_3_) afforded 464 mg of **12** (302 μmol, 84%) of as a colorless amorphous powder: ${\mathrm{[\alpha ]}}_{D}^{23}$ −26.7 (*c* 0.6, CHCl_3_); ^1^H-NMR (CDCl_3_, 0.83:0.13 mixture of rotational isomers) major isomer: 7.47–6.77 (35.69H, m), 6.91 (0.83H, d, *J* = 1.8 Hz), 6.73 (1.66H, s), 6.62 (0.83H, dd, *J* = 1.8, 8.0 Hz), 6.23 (0.83H, d, *J* = 2.4 Hz), 6.22 (0.83H, s), 6.14 (0.83H, d, *J* = 2.4H Hz), 5.82 (0.83H, t, *J* = 9.6 Hz), 5.18–4.82 (14.11H, m), 4.68 (0.83H, d, *J* = 9.6 Hz), 4.61 (0.83H, d, *J* = 10.2 Hz), 4.48 (0.83H, d, *J* = 10.2 Hz), 3.98–3.92 (0.83H, m), 3.57 (0.83H, s), 2.82 (0.83H, d, *J* = 17.2 Hz), 2.55 (0.83H, dd, *J* = 4.4, 17.2 Hz), 1.60 (2.49H, s), 1.51–1.50 (0.83H, m); minor isomer: 7.47–6.61 (5.98H, m), 6.62 (0.26H, s), 6.19 (0.13H, d, *J* = 2.4 Hz), 6.12 (0.13H, d, *J* = 2.4 Hz), 6.06 (0.13H, t, *J* = 2.4 Hz), 5.92 (0.13H, s), 5.18–4.47 (2.99H, m), 2.98–2.95 (0.26H, m), 1.50 (0.39H, s), a proton of hydroxyl group was not identified; ^13^C-NMR (CDCl_3_) 169.1, 158.2, 157.8, 156.6, 156.2, 155.8, 152.9, 149.1, 149.0, 137.9, 137.4, 137.3, 137.2, 137.0, 136.5, 128.7, 128.6, 128.4, 128.3, 128.2, 128.1, 128.0, 127.8, 127.7, 127.5, 127.3, 127.0, 121.3, 114.6, 113.6, 110.8, 108.3, 105.8, 100.2, 94.8, 94.3, 91.3, 80.4, 75.3, 73.0, 71.7, 71.3, 71.2, 71.0, 70.2, 70.0, 66.0, 35.2, 28.3, 20.7; ESILRMS (*m*/*z*); 1488 (42.8), 1487 (71.7), 1486 (67.2), 1472 (52.7), 1471([M + Na]^+^, 100), 1470 (94.3), 1449 (4.8), 1448 ([M + H]^+^, 4.8); ESIHRMS calcd. for C_95_H_83_O_14_, 1447.5783; found 1447.5777.

*(2R,2''R,3S,3''R,4S)-[4,8]-2,3-trans-3,4-trans-Nona-O-benzyl-(+)-catechin-(*−*)-epigallocatechin* (**20**). To a solution of **19** (184 mg, 127 μmol) in CH_2_Cl_2_ (20 mL) was added dropwise DIBAL–H (1.31 mL, 1.31 mmol, 1.0 M solution in hexane) at −78 °C. After stirring for 30 min at RT, the pale yellow reaction mixture was quenched with saturated Rochelle salt. The mixture was extracted with CHCl_3_ and the organic phase was washed with brine, and dried (MgSO_4_). Filtration, concentration, and preparative silica gel TLC purification (hexane/EtOAc, 2:1) afforded 176 mg of **20** (125 μmol, 99%) as a colorless amorphous powder: ${\mathrm{[\alpha ]}}_{D}^{23}$ −70.0 (*c* 2.06, CHCl_3_); ^1^H-NMR (CDCl_3_) 7.50–6.60 (50H, m), 6.22 (1H, s), 6.20 (1H, d, *J* = 2.8 Hz), 6.17 (1H, d, *J* = 2.8 Hz), 5.18–4.45 (20H, m), 4.28 (1H, t, *J* = 9.2 Hz), 3.91 (1H, br s), 3.77 (1H, s), 2.97 (1H, d, *J* = 17.3 Hz), 2.57 (1H, dd, *J* = 4.28, 17.3 Hz), a proton of hydroxyl group was not identified; ^13^C-NMR (CDCl_3_) 158.0, 157.8, 156.8, 156.1, 155.5, 153.3, 152.9 (×2), 149.3, 149.1, 138.0, 137.8, 137.4, 137.3, 137.2, 137.15 (×2), 137.08 (×2), 136.7, 134.3, 131.6, 128.6–127.1 (30C), 121.3, 114.8, 113.3, 111.9, 108.8, 105.6, 100.5, 94.9, 94.2, 91.4, 82.3, 75.2, 73.2, 71.3, 71.14 (×2), 71.06, 70.23, 70.16, 70.04, 69.98, 66.0, 36.9, 27.9; ESILRMS (*m*/*z*); 1446 (30.0), 1445 (47.6), 1444 (47.3), 1430 (41.0), 1429 ([M + Na]^+^, 77.4), 1428 (73.5), 1407 (12.3), 1406 ([M + H]^+^,16.8), 1405 (13.4); ESIHRMS calcd. for C_93_H_81_O_13_, 1405.5677; found 1405.5672.

*(2R,2''R,3S,3''R,4S)-[4,8]-2,3-trans-3,4-trans-(*+*)-Catechin-(*−*)-epigallocatechin* (**10**). A solution of **20** (140 mg, 99.6 μmol) in THF/MeOH/H_2_O (20:1:1, 22 mL) was added to the mixture and hydrogenated over 20% Pd(OH)_2_/C (5 mg) under H_2_ atmosphere for 12 h at RT. Filtration and concentration afforded a pale brown solid, which was purified with HPLC purification to give 12.6 mg of pure **10** (21.2 μmol, 21%) as a pale brown powder: ${\mathrm{[\alpha ]}}_{D}^{23}$ −138 (*c* 0.116, acetone) {lit. [29] ${\mathrm{[\alpha ]}}_{D}^{17}$ −168.9 (*c =* 1.0, acetone)}; ^1^H-NMR (CD_3_OD, 0.91:0.09 mixture of rotational isomers) major isomer: 7.04 (0.91H, d, *J* = 1.7 Hz), 6.90 (0.91H, dd, *J* = 1.7, 8.1 Hz), 6.85 (0.91H, d, *J* = 8.2 Hz), 6.73 (1.82H, s), 6.08 (0.91H, s), 5.88 (1.82H, s), 4.92 (0.91H, br s), 4.71 (0.91H, d, *J* = 7.7 Hz), 4.61 (0.91H, dd, *J* = 7.7, 10.6 Hz), 4.46 (0.91H, d, *J* = 10.6 Hz), 4.31–4.27 (0.91H, m), 2.93 (0.91H, dd, *J* = 4.1, 21.0 Hz), 2.85 (0.91H, d, *J* = 16.5 Hz); minor isomer: 6.79 (0.09H, d, *J* = 2.0 Hz), 6.70 (0.09H, d, *J* = 8.7 Hz), 6.59 (0.18H, s), 6.49 (0.09H, dd, *J* = 1.8, 8.4 Hz), 4.35 (0.09H, d, *J* = 4.7 Hz), 4.25 (0.09H, d, *J* = 6.6 Hz), 2.75–2.69 (0.18H, m), a proton of hydroxyl group was not identified; ^13^C-NMR (CDCl_3_) 177.5, 176.2, 175.0, 174.5, 173.9, 165.4, 165.0, 164.6, 152.0, 151.4, 150.6, 139.9, 135.3, 135.0, 126.9, 125.7, 118.3, 116.6, 116.5, 115.3, 102.5, 98.80, 92.52, 86.06, 57.35, 19.29; ESILRMS (*m*/*z*); 634 (7.8), 633 (23), 619 (10), 618 (42), 617 ([M + Na]^+^, 100), 595 ([M + H]^+^, 3.1); ESIHRMS calcd. for C_30_H_27_O_13_, 595.1452; found 595.1446.

### 3.3. DPPH Radical Scavenging Activity

DPPH radical scavenging activity was measured with general procedure [28]. A solution of DPPH radical in EtOH (30 μM, 1.0 mL) was added to 1 μL of the synthesized each compound in DMSO, and incubated at 30 °C for 30 min (*n* = 6). The scavenging activity was estimated with a microplate reader (Filter Max F5 multi-mode microplate reader; Molecular Devices, Downingtown, PA, USA) to measure the OD at 515 nm. Negative controls, the samples that 1 μL of DMSO added to the 1.0 mL of EtOH were prepared at the same time. And the absorbance values converted into the percentage radical scavenging activity as follows: [(absorbance of the control − absorbance of the sample)/absorbance of the control] × 100. VE (vitamin E) was used as the standard sample.

### 3.4. Antimicrobial Activity against S. cerevisiae

Overnight culture of *S. cerevisiae* was diluted to OD = 0.25 with culture medium, and 200 μL of aliquot was transferred to each well in a 96-well plate. Then 1 μL of the DMSO solution of compound was added in each well to a final concentration of 50 μM, and the growth of the *S. cerevisiae* at 27 °C with constant shaking was monitored by measuring absorbance at 600 nm every 15 min using an incubation reader, HiTS (Scinics, Itabashi, Japan). The results were obtained as growth curves as well as raw data. DMSO was used as negative controls. As negative controls, medium alone well and DMSO added to the well were prepared at the same time.

### 3.5. Inhibitory Activity of HeLa S3 Cell Proliferation

10^4^ cells per well with 100 μL of medium in a 37 °C incubator equilibrated with a 5% CO_2_: 95% humidified air atmosphere. D-MEM (Dulbecco’s Modified Eagle’s Medium; Gibco^®^ (Life Technologies, Grand Island, NY, USA) supplemented with 5% fetal calf serum and 1% Pen-Strep; Invitrogen™ (Life Technologies). After 24 h of incubation, 1 μL of synthesized six compounds in DMSO were added (final 100 μM) and incubated for 48 h. As negative controls, medium alone well and DMSO added to the well were prepared at the same time. After the medium was removed and the cell was washed with PBS, 90 μL of new medium and 10 µL of the MTT solution (3-(4,5-dimethylthiazol-2-yl)-2,5-diphenyltetrazolium bromide, 5 mg/mL) was added to each well and incubated at 37 °C for 2.5 h. After incubation the reaction medium was removed and 100 μL of DMSO was added to each well and mix thoroughly with the pipette. Following which, viable cells were assessed using a microplate reader (Filter Max F5 multi-mode microplate reader; Molecular Devices) to measure the OD at 570 nm.

## 4. Conclusions

In conclusion, we have synthesized four dimeric flavan-3-ol derivatives to clarify the importance of the upper-unit for biological activities. Our results support the assumption that the hydroxyl groups on the B-ring of the upper-unit are essential for the antimicrobial activity against *S. cerevisiae* and HeLa S3 cell proliferation inhibitory activity. Furthermore the correlation of antimicrobial activity against *S. cerevisiae*, proliferation inhibitory activity on HeLa S3 cells and of 2,2-diphenyl-l-picrylhydrazyl radical scavenging activity with the number of phenolic hydroxyl groups was low. Further synthesis and biological works to explain these mechanisms are now underway, since much more research using various analogs is needed to completely understand the SAR of these compounds.
